# Restoring AMs-HDAC1 expression in allergic asthma mice model by novel medicinal plant

**DOI:** 10.1186/2045-7022-3-S1-P2

**Published:** 2013-05-03

**Authors:** Lamia El-housseiny, Essam Hoaallah

**Affiliations:** 1 Medical University of Vienna, Universitatsklinik for Chirurgie; Kardiovaskulare, Austria; 2 National Research Center, Agriculture Microbiology Department, Egypt

## 

Worldwide **~**235 million people currently suffer from asthma. We searched the integrative medicine and the ancient cultures in the Middle East, Africa, and China for new anti-asthma treatment. LE25-0712 is an herbal extract (formula under patent). Here, we used it to validate the old knowledge known, to treat acute allergic asthma mice model. We also identify the effect of our novel extract on the expression of AMs-HDAC1 (alveolar macrophages-histone deacetylase1). We had proven in a previous study, deletion of HDAC1 had increased allergic airway inflammation and enhanced Th2 cytokine production in asthma mice model. In asthmatic and COPD patients, PBMC-HDAC level was reduced compared to healthy individual. In this study, we evaluated the effect of LE25-0712 on the expression level of AMs-HDAC1 in treated asthmatic mice (TAM) model compared to asthmatic control (AC) group.

## Methods

We used the acute asthma model of C57BL/6 mice, i.p. sensitized and i.n. challenged on d28, 29 with (OVA). LE25-0712 (0.5 mg/kg) was i.n. administered b.i.d (d25-29). WE assayed bronchoalveolar lavage fluid (BALF) for the total and differential types of inflammatory cells, and the expression of HDAC1 by IMF (d31). Näive and AC groups were included. As LE25-712 is a safe herbal supplement, allergic asthma patients were treated b.i.d by it combined with corticosteroid, until complete replacement of steroid.

## Results

LE25-0712 treatment of TAM group resulted in significant anti-inflammatory and anti- allergic activity as shown by reduced BAL total leukocytes (P < 0.03), and reduced eosinophils (P < 0.002) compared to the AC group. The expression of HDAC1 in AMs-näive was (87%), which was decreased in CA group to (8%). LE25-0712 restores HDAC1 expression in AMs of treated asthma model (45%, P < 0.0001). In 15 chronic asthmatic patients, LE25-0712 exerts a dramatic improvement in the general condition in 100% of patients, as it relieves the obstructive airway condition, which leads to complete spare of corticosteroids administration.

## Conclusion

The novel LE25-0712 is an effective natural safe medicinal extract for the prophylaxis of acute asthma disease in mice, as well as controlling the recurrent asthmatic attacks in chronic patients. The effectiveness and safety administration of this extract give it the upper hand over the traditional corticosteroid treatment. Moreover it restores the HDAC1 expression in TAM groups, which can be used as a follow up marker.

